# Germline mutations in *BRCA1* and *BRCA2* incidentally revealed in a biobank research study: experiences from re-contacting mutation carriers and relatives

**DOI:** 10.1007/s12687-017-0341-5

**Published:** 2017-10-30

**Authors:** Martin P. Nilsson, Monica Emmertz, Ulf Kristoffersson, Åke Borg, Christer Larsson, Martin Rehn, Christof Winter, Lao H. Saal, Yvonne Brandberg, Niklas Loman

**Affiliations:** 1 Division of Oncology and Pathology, Department of Clinical Sciences, Lund University, Lund, Sweden; 2 Department of Oncology, Skåne University Hospital, SE-221 85 Lund, Sweden; 3 Department of Clinical Genetics, Laboratory Medicine Region Skåne, Lund, Sweden; 4 Department of Clinical Genetics, Lund University, Lund, Sweden; 5 Department of Translational Cancer Research, Lund University, Lund, Sweden; 6 Department of Surgery, Skåne University Hospital, Malmö, Sweden; 7 Present Address: Institute of Clinical Chemistry and Pathobiochemistry, Klinikum rechts der Isar, Technische Universität München, Munich, Germany; 8 Department of Oncology-Pathology, Karolinska Institutet, Stockholm, Sweden

**Keywords:** Incidental findings, Biobank, Genetic, BRCA1, BRCA2

## Abstract

Once an incidental finding (IF) is discovered in the course of genomic research, the researchers are faced with the question of whether or not that finding should be reported back to the study participant. A large number of hypothetical studies and policy documents on this issue have been published, but there are very few empirical studies to inform the bioethics debate. Within a biobank research study of somatic mutations in breast carcinomas, ten germline BRCA1/2 mutations were incidentally detected. After thorough discussions within a group of experts, the mutation carriers (n = 7) or relatives of deceased carriers (n = 3) were re-contacted and informed about the findings. Eight out of ten accepted to receive the information and underwent confirmatory testing. One year later, semi-structured interviews were undertaken with three of the study participants. All of them felt that BRCA mutations discovered in the course of research should be reported back to the individual study participants. In this paper, we report our step-by-step experiences of the re-contacting process. We hope that our detailed reporting will be helpful for other researchers and clinicians that are faced with similar situations. The results of our study lend empirical support to opinion that IFs that meet the three baseline criteria of analytic validity, clinical significance, and actionability should be reported back to the individual study participants.

## Background

In the genomic research setting, an incidental finding (IF) is a finding concerning an individual research participant that has potential health or reproductive importance and is discovered in the course of conducting research but is beyond the aims of the study (Wolf et al. 2008). In the clinical setting, IFs are results that are not related to the indication for ordering the testing, but that may nonetheless be of medical value or utility to the patient (Green et al. 2013).

Over the last years, a large number of policy documents, debate articles, and hypothetical studies regarding the reporting of genomic IFs to study participants or patients have been published (Green et al. 2013; Fleming et al. 2015; Hehir-Kwa et al. 2015; Jelsig et al. 2015; Meulenkamp et al. 2010; JH et al. 2014). When reading such publications, a distinction between two different scenarios has to be made: first, whether laboratories performing DNA sequencing have a duty, or not, to actively seek and report IFs in genes not related to the indication for testing; and second, whether an IF already discovered in the course of research or clinical sequencing should be reported back to the individual of concern or not. For the first scenario, the debate is ongoing. For the second scenario, there is now broad consensus in the literature that IFs that meet the three baseline criteria of analytic validity, clinical significance, and actionability (ACA), such as germline mutations in the hereditary cancer genes *BRCA1* or *BRCA2*, should be reported back to the individuals (Knoppers et al. 2015). However, this consensus is almost solely based on the results of hypothetical studies and expert recommendations, which is problematic since the results of hypothetical studies might be poor predictors of actual decisions (Shkedi-Rafid et al. 2014). Furthermore, hypothetical studies are prone to sampling bias, and they might fail to convey the complexity of genetic risk information (Viberg et al. 2014). Very few empirical studies have been conducted to inform the bioethics debate regarding the reporting of IFs in high-penetrant genes (Hallowell et al. 2013; Haukkala et al. 2013; Ormondroyd et al. 2007; Richards et al. 2003). Some of the participants in the empirical studies experienced shock, anxiety, and denial upon learning of their mutation carrier status. Despite that, all or almost all of the interviewees in those studies felt that research results of importance should be reported back to study participants or relatives (Hallowell et al. 2013; Haukkala et al. 2013; Ormondroyd et al. 2007; Richards et al. 2003).

When mutations that meet the ACA criteria are discovered in a research setting, the researchers are confronted not only with the question of whether to re-contact and recommend the mutation carriers have follow-up in the clinical setting, but also practical questions such as: Who should make contact? How to contact? When to contact? Regarding these practical questions, the researchers currently will find very little guidance in the literature.

In this paper, we report our experiences from returning incidental *BRCA1/2* mutations detected within a research study of the All Breast Cancer in Malmö (ABiM) biobank cohort (Winter et al. 2016). The aim of the research study was to analyze by targeted exome sequencing the spectrum of somatic mutations in primary breast carcinomas at a single institution. For that purpose, germline DNA was sequenced and used to subtract germline variants. Incidentally, ten germline *BRCA1/2* mutations were found in individuals that were not aware of their mutation carrier status. Seven of them were alive and three were dead. At the outset of the ABiM biobank collection 10 years ago, the possibility of discovering highly penetrant germline variants that could potentially be reported back to study participants was not accounted for and the study participants had not been asked to consent to re-contacting, and were not informed about the potential for IFs.

After thorough consideration, we decided to re-contact the mutation carriers and, for deceased carriers, their next of kin. Here, we report our step-by-step experiences of the re-contacting process, from our motives to the participants’ views. We hope that our detailed reporting will be helpful for other researchers and clinicians that are faced with similar situations.

## Material and methods

### Study population

The study population has been described in detail elsewhere (Winter et al. 2016; Nilsson et al. 2017). Briefly, patients diagnosed with invasive breast cancer and scheduled for surgery during the years 2007 through 2009 in Malmö, Sweden, were asked prior to their surgery to participate in the population-based study ABiM and agree to donate a blood sample for research purposes and to consent the use of blood and tumor tissues for molecular and genetic analyses. Approximately, 80% of all invasive breast cancer patients in Malmö who were scheduled for surgery during the study period were included in the ABiM study (*n* = 538). The remaining 20% were either not asked (due to inability to understand written Swedish, psychological reasons, or other unspecified reasons) or declined to participate. No research tissue was taken unless it was certain not to influence the quality of diagnostic procedures. As a consequence, as well due to the quantity requirements of tumor and normal DNA and the quality requirements of sequencing data, 273 of the ABiM patients were analyzed within the somatic mutation screen study.

The aim of ABiM was to generate a biobank of patient material for translational research. The ABiM information sheet given to the patients at the time of breast cancer diagnosis did not include any information about hereditary breast cancer. Using this ABiM biobanked material, a mutation research study was performed with the aim to identify the spectrum of somatic mutations in breast carcinomas. For that purpose, germline DNA was used to subtract germline variants. The study participants consented to “*the blood sample and tumor samples will be stored and used for genetic studies and to measure certain substances in these samples*.” At the outset of the biobank collection, the possibility of discovering germline variants that should be reported back to study participants was not accounted for. Thus, the biobank participants had not been asked to consent to re-contacting and may not have been expecting to be re-contacted at a later point of time.

In the year of 2014, analyses of germline and tumor DNA, stored from time of diagnosis, were conducted. As previously reported, pathogenic germline mutations in *BRCA1* (*n* = 10) or *BRCA2* (*n* = 10) were detected in 20 patients (Winter et al. 2016). Because a clear medical benefit and supportive evidence for reporting IFs in the BRCA genes was significant by this time, but consent for re-contacting was not present, the question arose on how to confirm and proceed with the incidentally found germline mutations; should the mutation carriers be re-contacted or not?

### Process of re-contacting

In this section, the process of re-contacting is described. The re-contacting was performed according to the following protocol, which we developed after the IFs had been discovered and prior to any attempt to re-contact.

1. A literature search was performed of relevant empirical studies, hypothetical studies, policy documents, debate articles, and legislation, and over 300 articles or abstracts were reviewed.
2. An expert panel consisting of experts in oncology, clinical genetics, molecular genetics, biobank research, surgery, and genetic counseling met and discussed the procedure.
3. The Regional Ethical Review Board (ERB) was contacted in order to obtain approval to re-contact the mutation carriers. The ERB approved the ABiM study at the outset of the study in 2007 (Dnr 155/2007). They decided not to give a second approval for re-contacting, since they considered the question of re-contacting a clinical matter and not research.
4. We decided to re-contact the mutation carriers or next of kins (first-degree relatives) of deceased carriers. That decision was based on the fact that BRCA mutations, in our opinion, meet the criteria of analytic validity, clinical significance, and actionability. In addition, the mutation carriers had not explicitly taken a stand on not to be informed. For the mutation carriers, support for this standpoint was found in the literature. Little guidance was, however, found in the literature on the practical aspects of re-contacting, or how to deal with the next of kin for deceased carriers.
5. Approvals from the Heads of the Departments and Divisions of concern at Skåne University Hospital were obtained to re-contact the mutation carriers and next of kins of deceased carriers.
6. The medical records were reviewed and cross-referenced with the Onkgen database to identify which of the 20 cases who had, or had not, been tested within routine health care. All mutation carriers in the region are recorded in the Onkgen database. It turned out that ten of the mutation carriers had already been identified during the 5–7 years that had elapsed since their diagnosis of breast cancer. Accordingly, ten mutation carriers remained that had not been identified and were thus themselves not aware of their mutation carrier status. Seven of these were alive and three were dead.
7. To facilitate the re-contacting, it was decided to first thoroughly go through the medical records of the mutation carriers. The purpose was to identify carriers that for some reason should be re-contacted according to a special procedure instead of the standard procedure. Reasons for a special procedure could include inability to fully understand written Swedish or that a physician from the research team had been involved in the treatment of the patient. For deceased mutation carriers, the purpose was also to identify which relatives to contact. Beforehand, it was decided to first contact all children of the deceased mutation carriers. If there were no children, siblings were to be contacted, and if there were no siblings, spouses were to be contacted. The standard procedure of re-contacting is outlined below.
8. An invitation letter was sent to the mutation carriers (Appendix 1), stating: *“When analyzing your samples, we have discovered findings indicating that there could be a hereditary cause of your breast cancer. This might be of major importance for you and your health, but also for individuals in your family and other relatives. […] We would like to meet you and further explain these findings, and – if you wish to – confirm or reject our suspicion of a hereditary cause of your breast cancer through a complementary analysis. […] If you are interested in taking part of this information, we ask you to contact us per telephone, so that we can arrange an appointment at Skåne University Hospital.”*
9. A similar invitation letter was sent to the children of the deceased mutation carriers (Appendix 2)
10. The recipients of the letters were instructed to telephone a medical secretary at the Department of Surgery, Skåne University Hospital. This is a department that all of the ABiM study participants were familiar with, since they all had their primary breast cancer surgery there. Once they had called, an appointment was scheduled within 2 weeks. At that appointment, both an oncologist (NL) and a breast cancer surgeon (MR) attended.
11. A confirmatory blood test was taken during or after the appointment, and sent to a clinical lab for BRCA analysis.
12. Approximately 1–2 months after the initial appointment, the patient was invited to an appointment at the Department of Clinical Genetics, where a clinical geneticist (UK) and a genetic counselor attended. The patient was informed about the result of the confirmatory blood test. All mutations identified in the research study were confirmed. The families were then included in the established cascade screening program.
13. A reminder letter was sent to recipients that had not telephoned within 4 weeks. Thereafter, no further contacts were made.

### Semi-structured interviews

In May–June 2016, 1 year after the results of the confirmatory testing were given, three of the participants were contacted and invited for semi-structured telephone interviews. Two participants were not contacted due to not being fluent in Swedish. Two participants who had not been fully informed about the incidental findings were not contacted. Relatives of deceased participants were also not contacted for interviews. All three participants accepted to be interviewed. In the following, they are named AA, BB, and CC, respectively.

Interviews were conducted by a genetic counselor (ME) who had not been involved in the discussions preceding the re-contacting of the mutation carriers. The telephone interviews (duration 0.5–1 h) were semi-structured using a guide consisting of open-ended questions. The interviews were tape-recorded with consent and transcribed verbatim. Data analysis was conducted by two researchers (YB and MPN) according to the principles of IPA (interpretative phenomenological analysis), where themes from each transcript are used to construct super-ordinate themes (Ormondroyd et al. 2007).

## Results

Within a breast cancer biobank research study, ten germline mutations in *BRCA1* (*n* = 5) or *BRCA2* (*n* = 5) were incidentally revealed in study participants that were themselves not aware of their mutation carrier status, 5–7 years after they had been included in the study. At the time of re-contacting, seven of them were alive and three of them had died. In the following, “participants” refers to mutation carriers that were alive at the time of re-contacting, and “relatives” refers to the relatives of deceased mutation carriers that were contacted.

### ABiM study participants

In April–May 2015, six participants were contacted according to the standard procedure (see “Material and Methods” section). Four of them accepted the offer to come to an appointment following the invitation letter. One participant called and explained that she was not interested in any information, not specifying why. Another participant did not respond to the invitation letter or the reminder. We have no information on why she has not responded, but the address that the letters were sent to is most probably correct. In addition to these six participants, one participant was contacted according to a special procedure. She had previously attended genetic counseling at the Department of Clinical Genetics, but had not been tested. Therefore, she was contacted though an individual letter sent from the Department of Clinical Genetics and accepted the offer to come to an appointment.

### Relatives of deceased ABiM study participants

In October 2015, two daughters of two deceased mutation carriers were contacted according to our standard procedure (there were no other siblings in those families). One of them came to a visit following the invitation letter, and the other one came to a visit after a reminder. In addition to these two cases, the relatives of another deceased mutation carrier were contacted though a special procedure. This mutation carrier had been a patient of NL due to metastatic breast cancer, and NL therefore knew her husband well. NL contacted the husband and gave him a brief information about the mutation by telephone. More thorough information was revealed to him and his children at an appointment some weeks later.

### Interest in receiving the information

Altogether, we contacted ten individuals and offered them the information about incidental findings related to hereditary breast cancer. Eight out of ten accepted the offer to come to an appointment (seven following the invitation letter and one following a reminder). Two out of ten did not accept the offer to come to an appointment, and have therefore not been fully informed.

### Confirmatory analyses

All five participants who came to the first appointment had a confirmatory blood test taken at the first appointment or shortly after, and all the mutations were verified. For the deceased mutation carriers, relatives consented to confirmatory analyses in banked non-tumor tissue, and all three mutations were verified.

As we have previously reported, one of the deceased study participants was a carrier of the stop-gain variant *BRCA2* c.6901G>T in exon 12 (Winter et al. 2016). In the research analysis, this variant was classified as pathogenic, and it was thus reported back to the relatives of the participant. However, the clinical lab that performed the confirmatory analysis classified it as a variant of uncertain significance. Consequently, predictive testing for the relatives in that family has not been possible at this time, and the equivocal findings have caused some confusion for them. Functional analyses of the variant are now ongoing. All other mutations were classified as pathogenic by the clinical lab.

### Semi-structured interviews

Four super-ordinate themes emerged from analysis of the transcripts:

1. Reaction to the information letter.
2. Perceived advantages and disadvantages 1 year after the confirmatory testing.
3. Dissemination of the genetic information to family members and other relatives.
4. The process of re-contacting

## Reaction to the information letter

None of the interviewees had thought that the ABiM study was a study where one could be re-contacted in the future. The emotions and initial reactions to the information letter were mixed.

AA did not get scared: “I felt that I wanted more information about what it means for me and my children in the future. That was the strategy at once. Yes, I think it was.”

BB received the information in the letter with mixed emotions: “I got tired, I cannot take anymore now […] At the same time, I had the feeling that thank God it has been found out. Because now I have a chance to do something about it […] The soul and the head are not always in a match with each other. I was struggling between please, can it never end, and thank God I found out about it.”

CC was shocked: “I thought I donated a breast [tumor] for research and that was it, so I got a shock when I much later got called to all of this, because by then I had put all of that behind me.”

## Perceived advantages and disadvantages

One year after the confirmatory testing, all of the interviewees considered that there were more advantages than disadvantages with having received the information, although it had been a tough year, especially for CC. The decision on whether or not to undergo a prophylactic oophorectomy had rendered her much distress: “There has been a lot of stress […] It felt like a disfavor to start with, I thought much about it and so. But now as the process is over, it feels great”.

For AA and BB, it had been easier to adjust to their mutation carrier status. AA: “I think there are many advantages. One can check out who is a carrier, and take actions. My oldest daughter will participate in a surveillance program and it is taken seriously […] It is great to have been notified and I would not want to be without the knowledge.”

## Dissemination of the genetic information

AA and BB had informed all of their close relatives. Both of them felt that it had been harder for older relatives to receive the information that for younger relatives. BB had experienced some guilt:

“I think that one could feel some sort of guilt about that I have been ill. Because of my disease we have found out that I am a carrier of something. Now you have to test yourself. One could get some sort of feeling that I have infected you. Some guilt there. But that is not the way it is. No, absolutely not.”

For CC, it had been considerably harder to inform her relatives. She had informed her family, but not her cousins: “We have not been in touch since we were teenagers. It feels tough to call them so it has not been done […] There are so many thoughts. I feel that I have done what I can for me and my family, I do not have the energy to do any more.” After having informed her son, their relationship got worse: “It has not created a better relation with my son. I don’t dare to ask him if he has been to Lund [The Department of Clinical Genetics], but I don’t think he has.”

## The process of re-contacting

All of the interviewees were of the opinion that researchers should re-contact study participants to disclose important individual information, such as mutations in *BRCA1* and *BRCA2*.

BB: “I want to know everything that concerns me, I don’t want there to be a secret file about anything. I almost think that it is a bit unethical not to be able to take responsibility and decisions about one’s own health. So I would definitely want to know.”

CC: “People are different, but I did not think that I could be affected [a mutation carrier]. But it is good to have gotten the information, I believe so.”

None of them specified any better method for re-contacting than the method used. All three expressed, however, that they wished they could have been given the information sooner after their breast cancer diagnosis. Then the whole process of cancer and genetic predisposition to cancer would have been shorter. As it turned out now, the genetic information brought back some worries, not least the worry of cancer recurrence.

AA: “Imagine how different things would have been if one had been informed during the cancer treatment. The process would have been much shorter. It has taken many years of my life, treated and then sick again and now this.”

## Discussion

We report of a breast cancer research study of biobanked material where the participants had not been asked about whether they wanted to receive important individual information or not. Ten germline *BRCA1/2* mutations were incidentally detected in the course of research. After thorough discussions within a group of experts, it was decided to re-contact the mutation carriers (*n* = 7) or relatives of deceased carriers (*n* = 3). Eight out of ten accepted to receive the information and underwent confirmatory testing. We thought we could re-contact because there had been no specific mention of further contact, or not, at the time of consent.

To the best of our knowledge, our study is the first to report on disclose of incidentally found *BRCA1/2* mutations to biobank research study participants that have not explicitly consented to be re-contacted. Catenacci et al. reported on their experiences of re-contacting cancer patients who had undergone routine tumor sequencing for therapeutic intent, and had subsequently been identified as having an elevated risk of germline mutations (Catenacci et al. 2015). Despite some differences compared to our study, their results regarding the uptake of confirmatory testing resemble our results, with seven out of ten possible mutation carriers accepting the offer to undergo clinical genetic testing in their study (Catenacci et al. 2015).

In some other studies, legal and practical barriers have prevented the researchers from re-contacting individual mutation carriers. Keogh et al. and Pulford et al. reported of their experiences of providing aggregate information to study participants, i.e., no explicit individual information specifically to mutation carriers. Unsurprisingly, the uptake of confirmatory testing among the mutation carriers was lower with such an approach compared with our approach (Keogh et al. 2004; Pulford et al. 2016).

We were only able to interview three of the study participants, all of whom had undergone confirmatory testing. The results of the interviews must therefore be interpreted with caution. The themes that emerged from analysis of the transcripts are broadly in line with the results of previous studies (Hallowell et al. 2013; Haukkala et al. 2013; Ormondroyd et al. 2007; Richards et al. 2003). All of the interviewees felt that incidentally discovered BRCA mutations should be reported back to the individual study participants.

The wording in the invitation letter is most likely important. We did not write specifically about *BRCA1* or *BRCA2*, but “*When analyzing your samples, we have discovered findings indicating that there could be a hereditary cause of your breast cancer. This might be of major importance for you and your health, but also for individuals in your family and other relatives”* (Appendix 1–2). If the invitation letter would have been even more explicit, it is possible that the uptake of confirmatory testing would have been even higher. On the other hand, a more explicitly worded invitation letter would have taken away some of the freedom of choice that we aimed to retain.

We contacted The Regional Ethical Review Board (ERB) for guidance and approval to re-contact, as no consent had been obtained from the study participants for re-contacting. However, the ERB felt that the issue of re-contacting was not a matter of research, and therefore not in their domain of responsibility. We were somewhat surprised by this and do not agree with their standpoint. Furthermore, at the outset of the ABiM study almost 10 years ago, the ERB did not insist that individuals were asked to indicate whether they wanted feedback of important individual results. From now on, we believe that ERBs should require from researchers that are setting up biobanks to have a clear strategy on how to deal with incidental findings. Broad consents to feedback of important individual results is one alternative. However, it should be noted that broad consents are not without limitations; long consent forms could cause participants to feel overwhelmed and could create a therapeutic misconception (Shkedi-Rafid et al. 2014). Also, it has been questioned whether the choice of feedback in the consent form really enables participants to express what they truly prefer, since people might change their attitude to risk depending on what is at stake (Viberg et al. 2016).

In a recent survey of ERB professionals’ understanding of genetic IFs, only a minority (47%) of the respondents felt prepared to address them (Gliwa et al. 2016). This, as well as our experiences outlined above, highlights the need to educate ERB professionals about issues related to genetic IFs.

There are limitations to our study. First, there were only ten incidentally found mutations to disclose, and the low number precludes any subgroup or quantitative analyses. Second, these kinds of studies are always prone to selection bias. Richards et al. were able to interview 7 out of 14 mutation carriers (Richards et al. 2003), and Ormondroyd et at interviewed 13 out of 22 asked (Ormondroyd et al. 2007), but just like in our study, there is also selection bias in the steps prior to asking about any interest in being interviewed. For example, 80% of consecutive breast cancer patients were included in the ABiM study, but 20% were either not asked (due to inability to understand written Swedish, psychological reasons, or other unspecified reasons) or declined to participate. It is reasonable to assume that re-contacting patients such as the non-included ones might prove more difficult than what we report in this paper.

For the generalizability of our results, it is important to note that the implications of *BRCA1/2* mutations are more well-known and the evidence for clinical utility is more solid compared to mutations in genes with lower penetrance, such as *CHEK2*, or genes with more variable expressivity, such as *TP53*. Furthermore, due to the publicly funded and uniform health care system in Sweden, we experienced no problems with funding or questions regarding who should be responsible for the process of re-contacting. These could be important issues to consider in other countries.

When the issue of these IFs first appeared, it was not obvious to us what would be the best thing to do. In hindsight, we are content with our decision to re-contact the mutation carriers and the relatives of deceased carriers. As a consequence, members of seven families have been given the opportunity of participating in surveillance programs or opting for prophylactic surgery, and thereby substantially decreasing their risk of cancer-related deaths. These benefits have to be balanced against the possible negative psychological effects for the two participants who were not interested in receiving the information.

## Appendix 1: Invitation letter to study participant

Dear [name].

When you had surgery for breast cancer at Skåne University Hospital in Malmö a few years ago, you opted to participate in a research project named ABiM. As a part of that research project, you donated blood and tumor tissue for research purposes.

Through analyses of certain substances and genes in blood samples and tumor tissues, the aim of the research project was to explore new and better methods of treating breast cancer in the future.

When analyzing your samples, we have discovered findings indicating that there could be a hereditary cause of your breast cancer. This might be of major importance for you and your health, but also for individuals in your family and other relatives.

After having sought advice from ethical expertise, we now choose to contact you, to offer you a possibility to take part of this information. We would like to meet you and further explain these findings, and – if you wish to – confirm or reject our suspicion of a hereditary cause of your breast cancer through a complementary analysis. You are most welcome to bring a relative if you wish.

If you are interested in taking part of this information, we ask you to contact us per telephone, so that we can arrange an appointment at Skåne University Hospital. The appointment is free of charge for you.

The telephone number below goes to the secretary [her name] at the Section of breast diseases, Department of surgery at Skåne University Hospital in Malmö. She can put you in contact with us and schedule an appointment.

## Appendix 2: Invitation letter to deceased study participants’ daughter

Dear [name].

When your mother [name of the mother] had surgery for breast cancer at Skåne University Hospital in Malmö a few years ago, she opted to participate in a research project named ABiM. As a part of that research project, she donated blood and tumor tissue for research purposes. We have been informed that [name of the mother] has died, and we are sorry for your loss.

Through analyses of certain substances and genes in blood samples and tumor tissues, the aim of the research project that [name of the mother] chose to participate in was to explore new and better methods of treating breast cancer in the future.

When analyzing her samples, we have discovered findings indicating that there could be a hereditary cause of her breast cancer. This might be of major importance for her family members, i.e. siblings and children, but also more distant relatives.

The heritability of concern is such that there are possibilities of taking concrete prophylactic measures for relatives who might have inherited an increased risk of cancer.

After having sought advice from ethical expertise, we now choose to contact you, to offer you a possibility to take part of this information. We would like to meet you, together with other relatives if you wish, to further explain these findings, and – if you wish to – confirm or reject our suspicion of a hereditary cause of your mother’s breast cancer through a complementary analysis.

If you are interested in taking part of this information, we ask you to contact us per telephone, so that we can arrange an appointment at Skåne University Hospital in Malmö. The visit is free of charge for you.

The telephone number below goes to the secretary [the secretary’s name] at the Section of breast diseases, Department of surgery at Skåne University Hospital in Malmö. She can put you in contact with us and schedule an appointment.
